# Fishing for Catalysis: Experimental Approaches to Narrowing Search Space in Directed Evolution of Enzymes

**DOI:** 10.1021/jacsau.3c00315

**Published:** 2023-08-18

**Authors:** Liam R. Marshall, Sagar Bhattacharya, Ivan V. Korendovych

**Affiliations:** Department of Chemistry, Syracuse University, 111 College Place, Syracuse, New York 13224, United States

**Keywords:** directed evolution, protein dynamics, catalysis, enzymes, mutagenesis

## Abstract

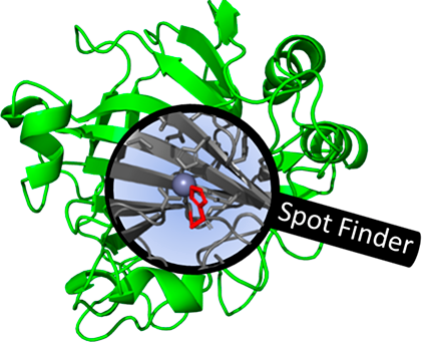

Directed evolution has transformed protein engineering offering a path to rapid improvement of protein properties. Yet, in practice it is limited by the hyper-astronomic protein sequence search space, and approaches to identify mutagenic hot spots, i.e., locations where mutations are most likely to have a productive impact, are needed. In this perspective, we categorize and discuss recent progress in the experimental approaches (broadly defined as structural, bioinformatic, and dynamic) to hot spot identification. Recent successes in harnessing protein dynamics and machine learning approaches provide new opportunities for the field and will undoubtedly help directed evolution reach its full potential.

## Introduction

**Figure 1:**
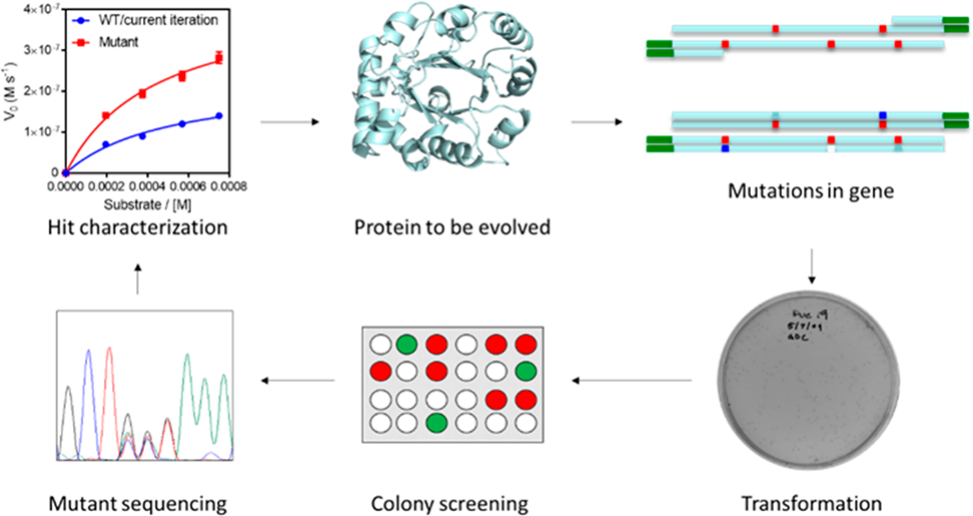
Overview of directed evolution. Mutations are introduced into a gene encoding the protein of interest, followed by transformation into an organism, which is selected for survival or screened for function. The hits identified in the screening/selection are characterized in detail and serve as a template for the next round of evolution.

Directed evolution is a powerful technique to evolve proteins’ properties or impart complete functionalities onto them. This technique relies on introduction of mutations into a protein and subsequent screening and selection for the improved property (enzymatic efficiency, selectivity, stability, etc.) that can be done iteratively. By testing even a relatively small number of residues, remarkable improvements in the desired outcome can be achieved. Since the early seminal studies by Hageman and Arnold on improving thermostability of enzymes, it has been expanded to other important properties.^1,2^ Even for evolving such complex functionality as catalytic activity, which requires concerted action of multiple residues through multiple steps along the reaction coordinate, very impressive achievements can be made.^3^ In the most simplistic form directed evolution involves preparation of a DNA library, in the early examples using error-prone PCR (epPCR), that is then transformed into the organism of choice (typically *E. coli*) followed by selection or screening for improvement in the desired property (Figure 1).^4^

Screening for the activity is most often the limiting step in the overall approach as only high-throughput spectroscopic approaches or survival selection methods would allow one to sample the number of variants that can get close to transformation efficiency, which scales linearly at about 10^8^ variants per 20 μg of plasmid DNA (∼10^13^ DNA molecules) per 1 mL of cell culture. The most efficient strategy for high-throughput screening currently available can screen 10^8^ variants per day.^5^ While quite large (and can be even larger by using mRNA display techniques),^6^ this impressive number pales in comparison with the number of reasonable options to test in a typical protein. A typical enzyme is comprised of ∼250 residues (as found in the TIM barrel fold) yielding 20^250^ possibilities, a hyper-astronomical number of possibilities that vastly exceeds the number of particles in the universe (estimated to be “only” on the order of 10^80^). Obviously, not all possible options need to be tested in a directed evolution experiment, but ∼10^11^ possibilities cover just about 10 positions to be simultaneously fully tested, a number clearly insufficient in a large protein. And that assumes perfect DNA libraries with full coverage and no redundancies, not something even remotely achievable in practice, due to imperfect PCR amplification, primer quality, and fundamentally, degeneracy of the genetic code.

Given the effort involved in increasing screening efficiency and throughput, it was only natural that since the early days of the approach much effort was directed toward narrowing the sequence search space in directed evolution experiments to be feasibly experimentally evaluated. Directed evolution has been used to improve different protein properties (notably, thermostability and ability to work in organic solvents), but in this perspective, we will provide outlook on experimental approaches to hot spot determination in the directed evolution of enzymes to improve their efficiency and selectivity, arguably the most complex task, with an, by no means exhaustive, overview of a few representative examples, comparing their advantages and disadvantages and our thoughts on the future of the method.

There are thousands of papers published on the directed evolution of enzymes with multiple complex and elaborate optimization approaches, yet their underlying principles are still quite simple, and we broadly characterize all of them as structural, dynamic, and bioinformatic.

## Structural Approaches to Identifying Mutagenic Hotspots

**Figure 2:**
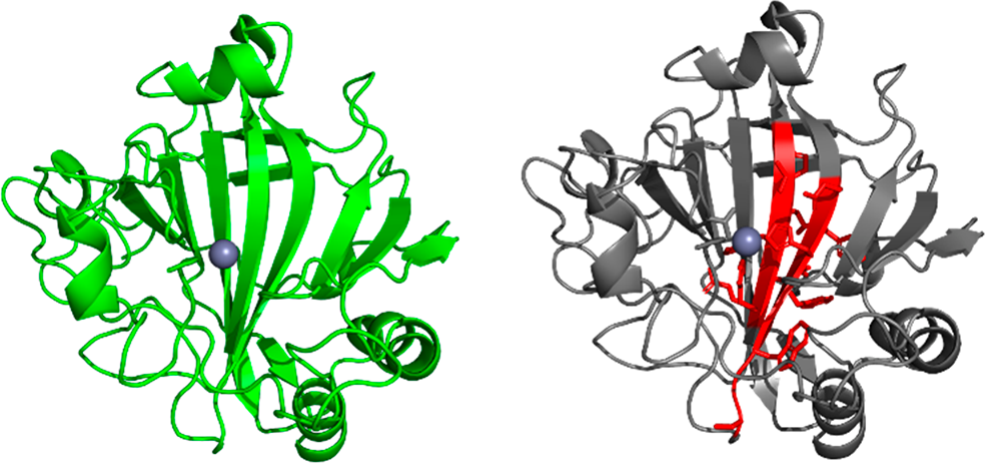
The structural approach to hot spot identification. Available structural information serves as a basis for rational identification of residues (shown in red) likely to influence activity (based on proximity to the active site, substrate tunneling, etc.).

The wealth of structural information available through the PDB, and recently, through machine learning approaches, provides ample opportunities for rational and semirational guidance of directed evolution experiments. It was recognized early on that the probability of finding function altering mutations is highest in and around the active site (Figure 2). In a way, this approach traces back all the way to Emil Fischer’s lock-and-key hypothesis^7^ and its expansion by Linus Pauling.^8,9^

**Figure 3:**
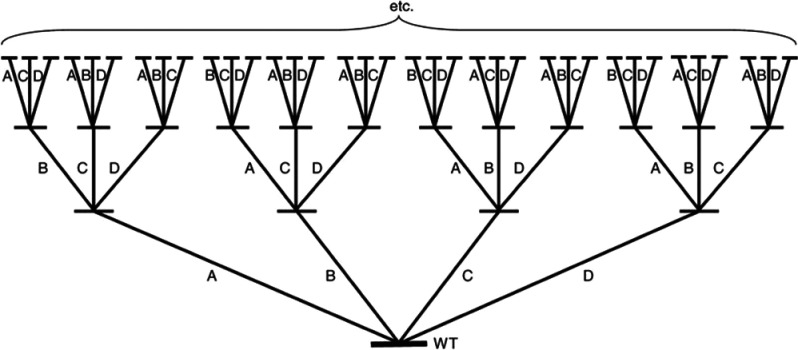
Iterative saturation mutagenesis overview. Saturation mutagenesis in four positions (A, B, C, D) is performed iteratively to improve sampling efficiency to catch the synergy between residues. Reproduced with permission from ref (15). Copyright 2007 Springer Nature.

**Figure 4:**
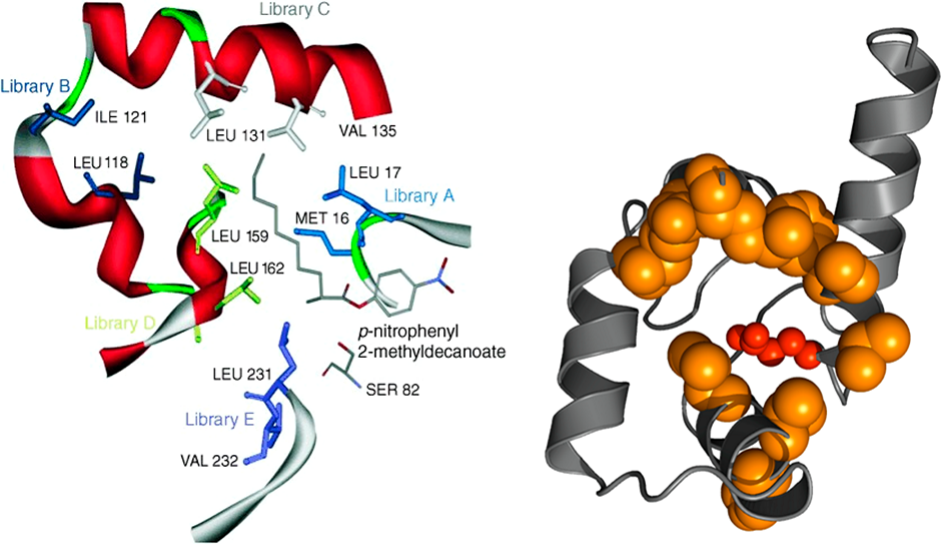
(Left) Illustration of CAST library design. Design of five libraries of mutants (A–E) produced by simultaneous randomization at two amino acid sites in the lipase from *Pseudomonas aeruginosa* using CAST. The docked substrate illustrates the position of the active site and the substrate binding pocket. Reproduced with permission from ref (21). Copyright 2005 Wiley-VCH. (Right) Residues chosen for structure-based evolution of AlleyCat (shown as orange spheres) located in the proximity of the active Glu92 residue (shown in red).

In a classic series of papers, Reetz and co-workers first demonstrated in 1997 that the typical by then epPCR approach can work to alter stereoselectivity of a *Pseudomonas aeruginosa* lipase (PAL) by about 10-fold.^10^ This was a major advance, demonstrating practical utility of directed evolution to create industrially relevant enzymes. Yet epPCR quickly exhausted its potential and its further application yielded negligible stereoselectivity improvement. However, application of saturation mutagenesis, where all 20 amino acid residues are tested in a single position, to the substrate binding site in an approach termed combinatorial active site saturation test (CAST, Figure 4) allowed for rapid further selectivity improvement to more than 51-fold.^11^ Next, using structural information and iterative saturation mutagenesis (ISM, Figure 3), where the residues chosen in the active site are tested iteratively providing insight into potential synergistic interaction without testing the whole combinatorial space, Reetz and co-workers were able to further improve stereoselectivity to reach an extremely high ratio of ∼600.^12^ In another major contribution to the field Reetz demonstrated that limited amino acid libraries, e.g., using the NDT codon that covers 12 amino acids, representing most chemical functionalities, saturation mutagenesis in the active site can be practically applied to multiple residues simultaneously, providing a good view of the full fitness landscape and further improving the efficiency of the method.^13^ Interestingly, amino acid alphabets can be limited drastically to even smaller alphabets (typically 5–7 residues), offering full combinatorial coverage in many positions simultaneously, possibly covering the full substrate binding site, with good results.^14^

Structural approaches to hot spot identification have been particularly impressive in repurposing highly active and promiscuous enzymes, such as cytochrome P450 (CYP450) family enzymes, to promote stereoselective substrate oxidation and even repurpose the enzyme to catalyze other types of organic transformations, not present in nature. For example, using ISM in the substrate binding pocket Li and co-workers converted cytochrome P450 hydroxylase (p450pyr) into a highly enantioselective hydrolase for *N*-benzyl pyrrolidines.^16^ Using ISM of the residues in the substrate binding channel (as determined by computational docking of the substrate into a crystal structure of the enzyme, Figure 4) aided by ISM, Li and co-workers were able to evolve p450pyr into a highly efficient stereoselective subterminal hydroxylase.^17^ The same approach with very impressive results was applied to CYP450 BM3, the enzyme with the reductases genetically fused to ensure constant turnover by adding NADH, to create highly regioselective hydroxylases with multiple stereocenters formed in one step.^18,19^

**Figure 5:**
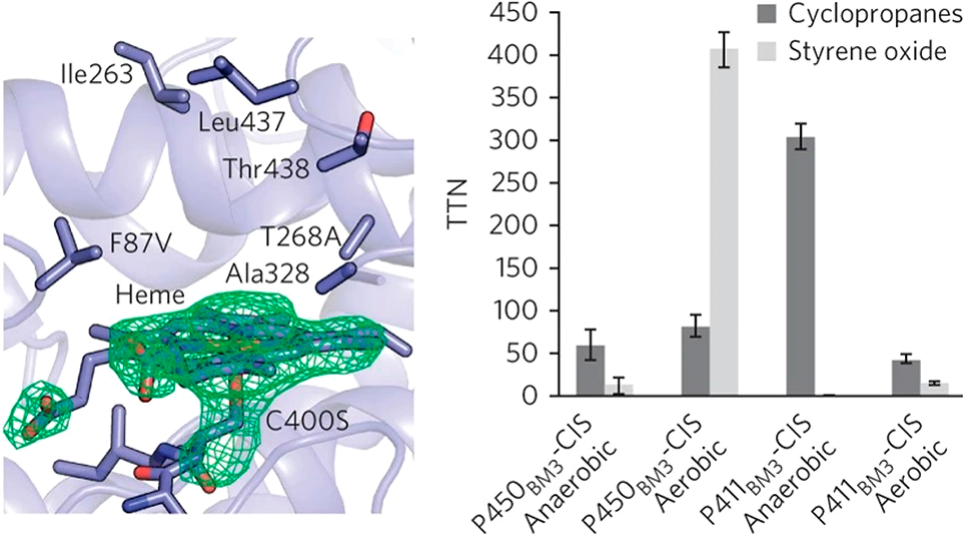
Active site of cytochrome P450 engineered to promote cyclopropanation (P411_BM3_-CIS). (Left) Close up of the P411_BM3-heme_-CIS active site showing the mutations introduced (C450S, F87 V, T268A) as well as other residues important for catalysis. (Right) In vitro cyclopropanation versus epoxidation of styrene for different P450 variants. Reproduced with permission from ref (24). Copyright 2013 Springer Nature.

Impressive selectivity for complex clinically relevant substrates has also been achieved by mutating residues in the active site of CYP450 followed by high-throughput screening (Figure 5).^20^ Efforts by many laboratories resulted in large libraries of mutants providing sequence-function relationships, and quickly enough, it was recognized that some positions and even individual mutations show disproportionally high propensity to promote new reactivity driving semirational evolution for other substrates, which is especially valuable for the reactions that are difficult to screen for in a high-throughput fashion.

Arnold and co-workers realized the similarity between compound I, the ultimate oxidant in cytochrome P450, and carbene and nitrene species. This allowed them to expand the repertoire of the reactions promoted by these enzymes to “unnatural” reactions. By screening large libraries of P450 BM3 mutants, they identified several that showed promising activity in cyclopropanation^22^ as well as C–N bond formation using diazo compounds (Figure 5).^23^ Further mutagenesis around the active site and rational modulation of the metal cofactor redox potential by replacing axial cysteine with serine allowed for significant improvement in activity and selectivity.^24,25^

Realizing that the power of directed evolution can repurpose other heme protein scaffolds to perform various oxidative transformations without relying on a fairly complex CYP450 system, Fasan and co-workers reshaped the oxygen binding pocket of myoglobin to accept a variety of different organic substrates and efficiently promote C–H functionalization.^26^

Purely structural approaches can be particularly powerful in the directed evolution of *de novo* designed proteins, especially those designed using the minimalist approach in fairly “naïve” scaffolds, where the initial activity is low and the active site is not well adapted for the reaction of interest.

We have shown that basic computation tools can be used to introduce a single mutation into calmodulin to convert this nonenzymatic protein into a lyase, with 5 orders of magnitude activity improvement of the background rate.^27^ Saturation mutagenesis of the residues lining the active site (Figure 4) identified productive mutations in 4 positions, producing a *k*_cat_/*K*_M_ improvement of 7.5-fold.^28^

**Figure 6:**
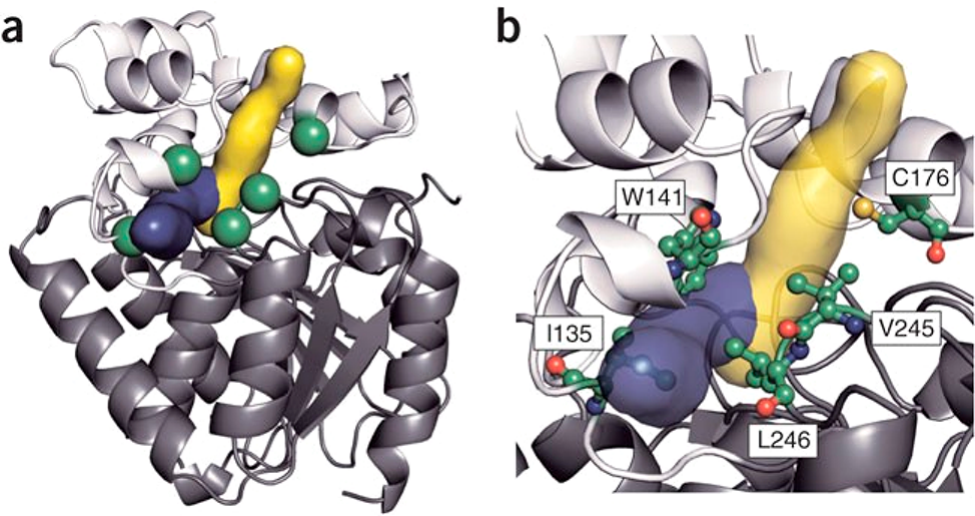
Enzyme engineering by targeting access tunnel residues. (a) Cartoon model of wild-type DhaA. Gray, main domain; white, cap domain; yellow, product release pathway corresponding to the main tunnel; blue, product release pathway corresponding to the slot tunnel; green, residues selected for mutagenesis. (b) Ball and stick model of the residues selected for mutagenesis. Reproduced with permission from ref (30). Copyright 2009 Springer Nature.

Structural approaches to hot spot identification are not limited to active sites alone and have successfully been used to reshape substrate channels, leading to buried active sites.^29^ Damborski and co-workers engineered haloalkane dehalogenase DhaA to convert a toxic 1,2,3-trichloropropane (TCP) into a less toxic 2,3-dichloropropan-1-ol (DCP).^30^ Structural analysis of DhaA using CAVER, a computational tool developed to identify channels in protein structures, was used to localize the substrate access (Figure 6).^31^ Then residues in five different areas of the substrate channel were subjected to saturation mutagenesis in two libraries, yielding a quintuple mutant with a 26-fold improvement in activity.

Overall, the structural approaches have been well established for two decades or so and are still used extensively, especially for altering enzyme specificities, where reshaping the active site alone can lead to quick results. Thus, it is often applied prior to other methods. It has been particularly powerful when reshaping the active site to alter enzyme specificity and extend them to other substrates. Given the early success of the method, it was only natural that purely structural approaches were combined with computational tools, such as docking, molecular mechanics (MM) tools, etc., to further improve efficiency of finding the beneficial mutations, and currently most examples of structure-based directed evolution include some computational aspects. Substrate and transition state geometries using even basic docking studies can be established with good accuracy, highlighting interactions essential for catalysis. Moreover, combining CAST with MD simulations and ML approaches can help pinpoint possible identities of the productive mutations.

On the other hand, while enormous progress has been made in structure characterization and prediction for well-folded proteins in the past decade, with AlphaFold2 making high quality structural predictions almost routine, this information is inherently static and, while very useful, alone does not provide full guidance to rationally guide directed evolution. Purely structure-based approaches have a hard time predicting hot spots far from the active site and its immediate vicinity. Such allosteric interactions can drastically alter functionality of the enzyme through mostly dynamic interactions, although other interaction modalities (electrostatic, etc.) have been implicated as well.^32^ Although, it should be noted that sequence information and MD simulations together can provide some predictions regarding allosteric interactions.^33^ The structural approaches work best when combined with computational techniques, as structure gazing alone has its limitations, which often requires additional expertise, collaborations, and may be more time-consuming.

## Bioinformatic Approaches to Hot Spot Identification

**Figure 7:**
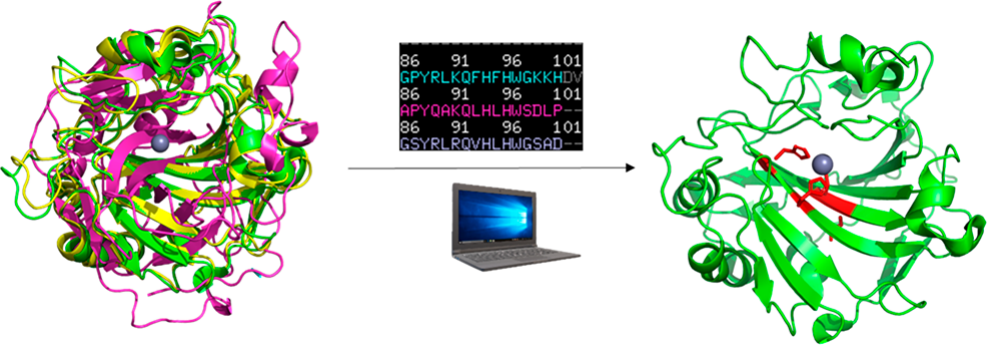
The bioinformatic approach to hot spot identification. Bioinformatics approaches rely on analysis of large sequence data sets to identify common and useful structures within a protein which contains the desired functionality.

The advent of efficient low-cost sequencing strategies resulted in a huge number of protein sequences available (177 million sequences in UniProt alone).^34^ Aligning enzyme sequences from different isoforms or organisms using multiple sequence alignments (MSA), even in the absence of structural data, can provide valuable information about the role of different residues in catalysis (Figure 7). MSA have extensively been used in protein engineering to improve enzyme stability, catalytic activity, and enantioselectivity,^35−38^ particularly when structural and/or functional information is limited. The idea behind this approach is that varying residues that are not universally conserved, but have different (CBD) sites, offers a quick path to varying specificity and improving activity of the enzyme, using identities of the residues determined in the MSA.

**Figure 8:**
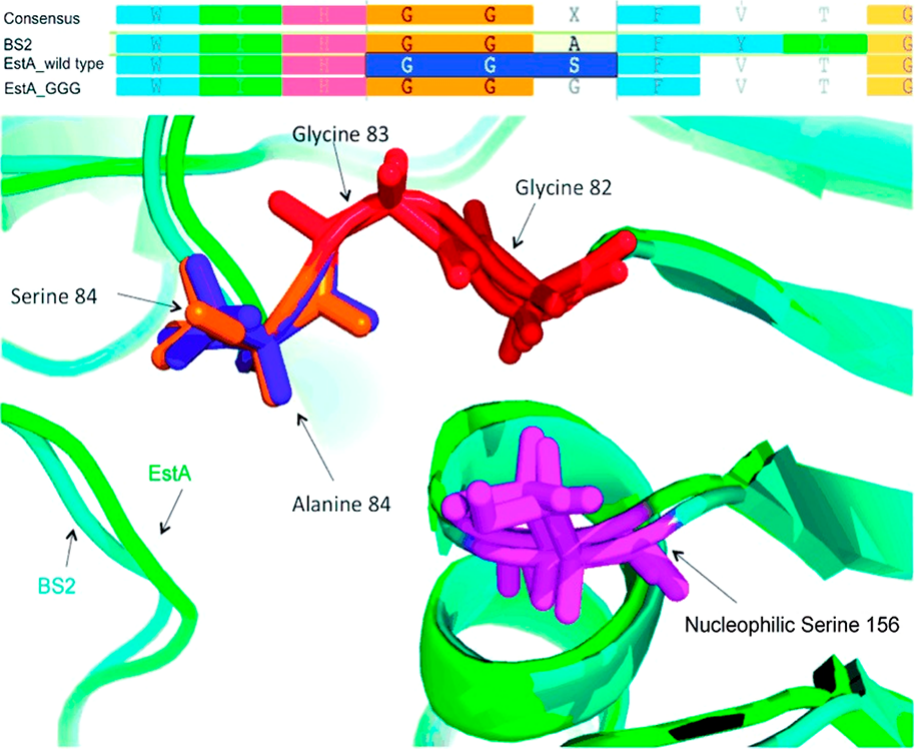
Hot spot identification by multiple sequence alignment. Active site of GGX esterases. (Top) Alignment of the oxyanion hole region of BS2, EstA, and the consensus of the 1343 sequences of the acetylcholine esterase-like enzyme family. (Bottom) Active site of esterase BS2 (cyan) and the homology model of EstA (green). The catalytic nucleophile and the GGX motif are highlighted as sticks. Reproduced with permission from ref (39). Copyright 2010 Wiley-VCH.

In a classic example, Bornscheuer and co-workers evolved *Paenibacillus barcinonensis* esterase (EstA) to hydrolyze tertiary alcohol esters. EstA hydrolyzes tertiary alcohol esters with limited activity and enantioselectivity, yet its sequence homologues promote its reaction efficiently. MSA analysis of 1343 EstA homologues revealed a highly conserved GGX motif in the oxyanion hole, where X is a small residue (Figure 8). Mutating the serine in the GGS sequence in *P. barcinonensis* EstA to glycine led to a 26-fold improvement in ester hydrolysis over the wild type.^39^

Additionally, larger bioinformatic data sets allow for correlating identities of the allosteric sites though analysis of covariation of residues in large data sets. Residues which are only conserved in the enzyme subfamily may be indicative of their significance in functional diversification.^40−42^ Some of these residues often act complementarily, particularly in terms of size and/or charge. These coevolutionary couplings often fine-tune the properties of enzymes, including stability and catalytic activity. This evolutionary approach, dubbed evolutionary coupling saturation mutagenesis (ECSM) can be productive in improving properties of existing enzymes. For instance, seven residue pairs identified in ECSM analysis of the pullulanase from *Bacillus naganoensis* were selected for saturation mutagenesis that led to a quadruple mutant exhibiting 3-fold improvement in *k*_cat_ and 6-fold enhancement in catalytic efficiency (*k*_cat_/*K*_M_) over wild type.^43^ Apparent activity improvement can be achieved by introducing consensus mutations from thermostable analogs of the protein of interest. For example, Baker, Tawfik, and co-workers increased the activity of KE59, a computationally designed Kemp eliminase, by introducing residues found in thermophilic analogs of indole-3-glycerolphosphate synthase that served as a basis for the design. By spiking consensus mutations, they stabilized the mostly unfolded protein enough to achieve the structure that supports the initial computation design with at least a 2-fold increase in measured activity.^44^

**Figure 9:**
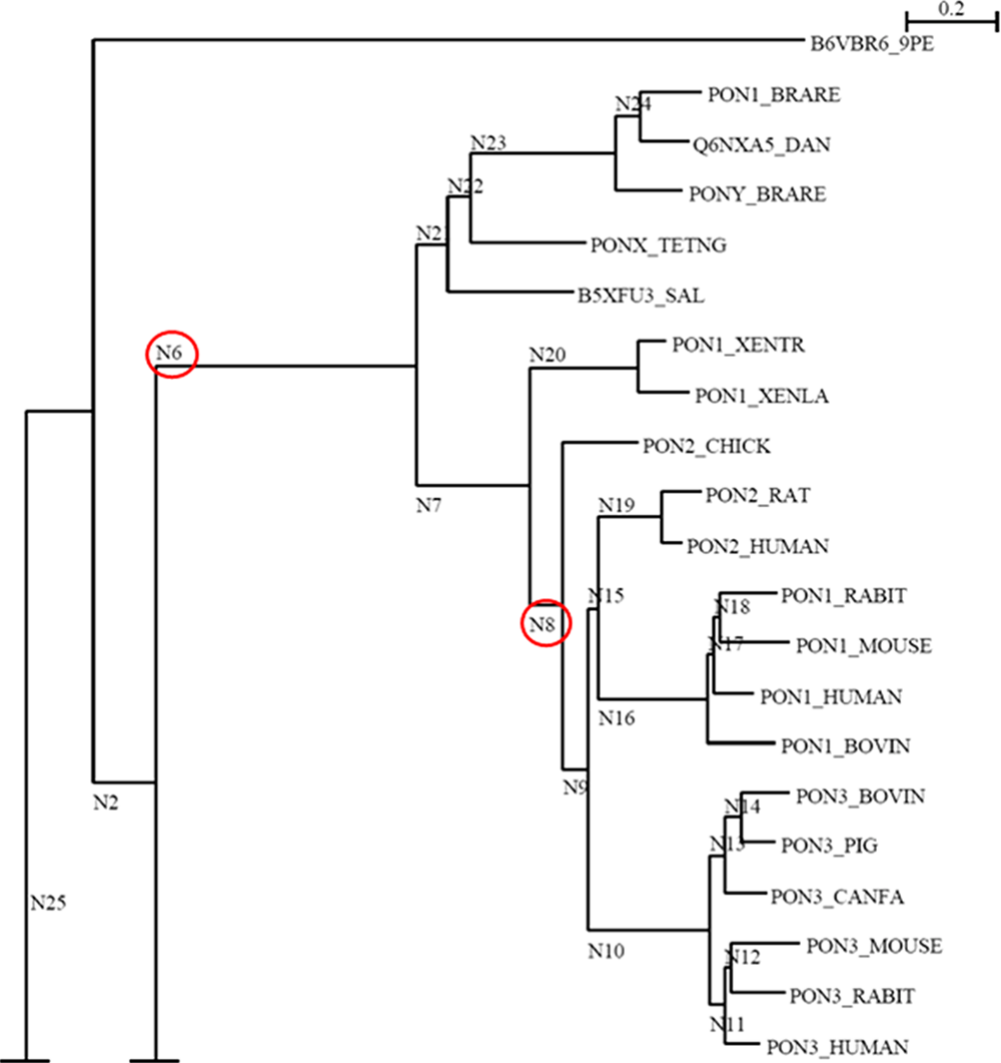
Phylogenetic trees to guide catalysis. A phylogenetic tree of the PON family, where the nodes represent various ancestors. N6 is an ancestor predicted for all vertebrate PONS, while N8 is the ancestor of mammalian and chick PONs. Not shown are the bacterial sequences (N25) that show ∼30% sequence similarity to vertebrate and mammalian PONs which served as an out-group. Adapted with permission from ref (45). Copyright 2011 Elsevier.

The bioinformatic approach can be extended to include complex evolutionary relationships. On the basis of the notion that evolutionary early enzymes possessed higher stability and promiscuity, phylogenetic analyses to identify sequences of “primordial” enzymes can be generated and experimentally tested. Tawfik et al. used serum paraoxonases and cytosolic sulfotransferases as the model enzymes for ancestral library design to improve the catalytic efficiency of enzymes (Figure 9). Both the enzyme families play a critical role in drug metabolism and detoxification of xenobiotics. Mammalian serum paraoxonase (PON) includes enzyme families PON1, PON2, and PON3 that share 55–85% sequence identity. These calcium-dependent enzymes are capable of hydrolysis of esters, lactones, and phosphoesters. Orthologs and paralogs of the PON family were selected for sequence alignment to create a phylogenetic tree, which was used to assign the most probable sequences for ancestral nodes. The ancestral node N8 for PON enzyme families (N8-PON), which had high reliability in sequence prediction, was chosen for library design. Library screening yielded at least 12 variants that showed activity improvement ranging from 2- to 12-fold in hydrolyzing paraoxon over the starting PON1 variant. This strategy was extended to cytosolic sulfotransferase family (SULT) as well, and construction of ancestral library on N8 node of SULT1A1 (wild type) identified 16 positions as hot spots for subsequent screening. Library screening identified the SULT1A1-b9 mutant with about 6-fold improved activity for 3-cyanoumbelliferone. Crystal structure of the improved variant also shows that F247I mutation at the active site assists in opening up the binding pocket to accommodate larger substrates, underscoring the role of ancestral mutations in catalysis.^45^

Ancestral reconstruction can also be used prior to design to increase stability and evolvability. Sanchez-Ruiz and co-workers took advantage of the high stability of Precambrian β-lactamases to introduce novel functionalities. The loop region (residues 225–229) of GNCA was identified as a suitable target for enzyme engineering considering the possibility of conformational reorganization. A single mutation W229D enabled the enzyme to show distinguishable Kemp eliminase activity over the background. Although no new active site was generated as a result of this mutation, conformational rearrangement allowed this hydrophobic-to-polar replacement with Asp229 to serve as the catalytic base for the transformation. The best Precambrian variant GNCA4-W229D/F290W catalyzes Kemp elimination with a catalytic efficiency of 5000 M^–1^ s^–1^,^46^ which subsequently was improved by 4-fold to 20,000 M^–1^ s^–1^ using FuncLib.^47^ Further improvement of this approach yielded a highly active enzyme with a catalytic efficiency of 200,000 M^–1^ s^–1^.^48^

## Machine Learning in Directed Evolution

**Figure 10:**
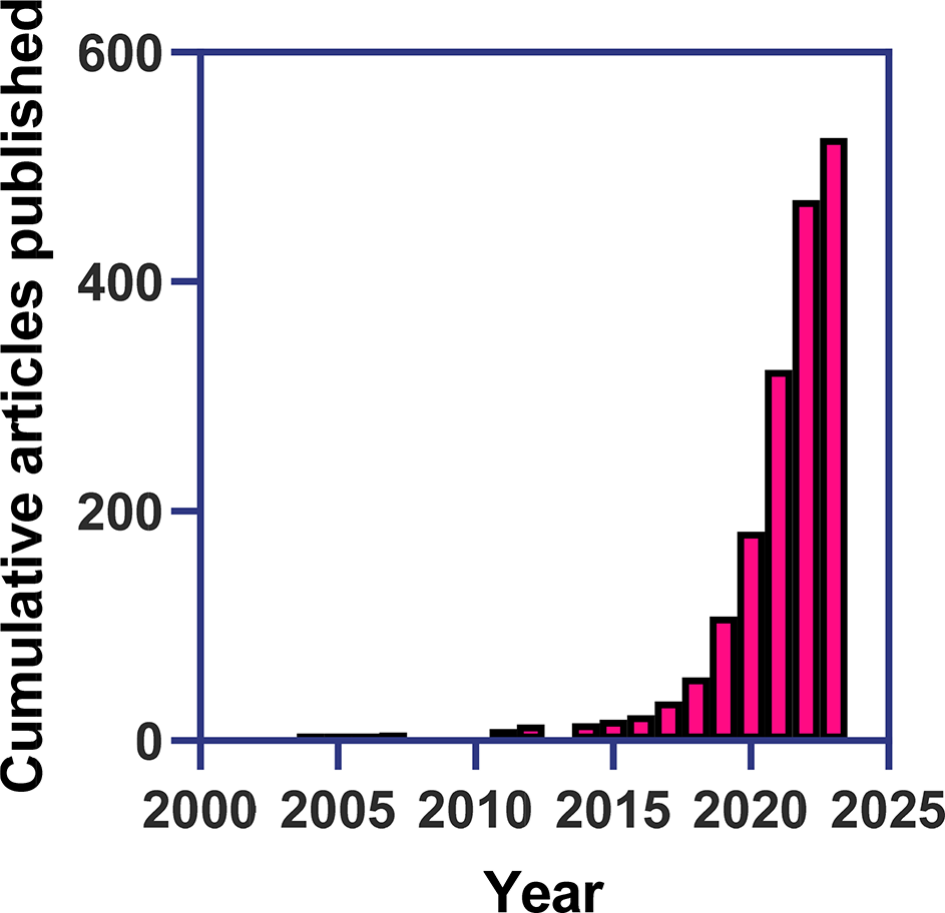
Cumulative number of papers published with keywords “machine learning” and “catalysis”.

Machine learning approaches have taken the world by storm, and life sciences have not been an exception. Ever since the spectacular success of AlphaFold and its successor AlphaFold2,^49,50^ there has been a lot of interest (and high expectations) in applying machine learning methods to protein engineering and specifically to directed evolution. In Figure 10, we show the exponential growth of published papers devoted to machine learning studies for catalysis with a turning point happening around 2017. Since this approach productively combines large data sets, we view it as fundamentally bioinformatic and thus place it into this category. Several recent reviews provide an excellent introduction into this rapidly growing field,^51,52^ so here we will just provide several representative studies.

Arnold and co-workers used machine learning to reduce the experimental cost in directed evolution. Looking to minimize mutant screening using traditional directed evolution techniques, they utilized machine learning-assisted directed evolution in their efforts to evolve an enzyme to produce each of the two possible product enantiomers of carbene Si–H insertion, a new-to-nature reaction and an excellent test of their approach.^53^ They first trained their machine learning model on an empirical fitness landscape for human GB1 binding protein associated with an antibody, which provided a large experimental data set. Next, they applied it on a putative nitric oxide dioxygenase to create two enzymes capable of producing the R and S-enantiomers, with 93% and 79% enantiomeric excess (*e.e*.), respectively. Machine learning served to sample combinatorial libraries of mutants, effectively allowing their model to predict multiple beneficial mutations *a priori*, rather than the individual stepwise mutations directed evolution traditionally relies on.

**Figure 11:**
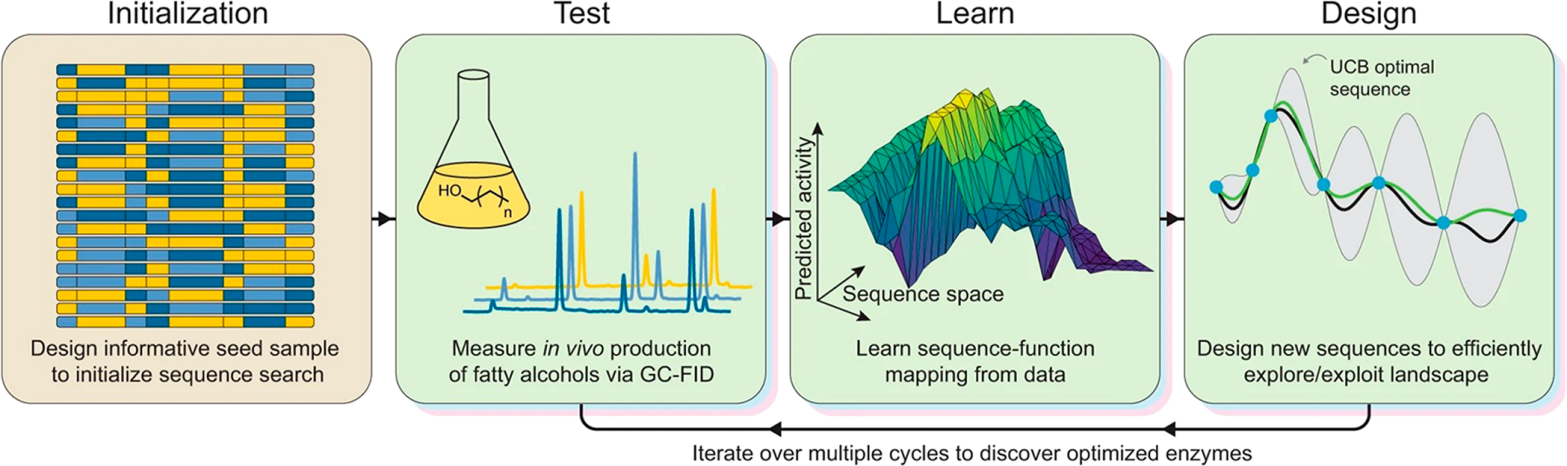
Overview of ML-assisted directed evolution. The search is initialized by designing a diverse set of constructs that broadly sample the sequence landscape. Multiple design-test-learn cycles are then employed to optimize in vivo fatty alcohol production. Reproduced with permission from ref (54). Copyright 2021 Springer Nature.

Romero, Pfleger, and co-workers used a ML-assisted approach to improve the activity of alcohol-forming fatty acyl reductases (FARs) to produce fatty alcohols from intracellular metabolites in vivo (Figure 11).^54^ Using iterative cycles of representative sampling (no more than 20) of sequence blocks derived from difference organisms in 8 regions of acyl–acyl carrier domain (ACR), the authors were able to quickly converge onto the optimal sequence, without the need for a larger investigation of all 4374 possibilities. The improved enzyme produces approximately double the amount of alcohol as compared to the original chimera. This work establishes ML learning algorithms that can be ultimately expanded to the shorter, ultimately single residue, sequence fragments. The sequence-based bioinformatic approach can quite rapidly identify areas for productive mutations. In many cases, it also provides identities of the possible mutations that drastically cut down on the number of reactions to be screened and allows for the use of low-throughput characterization techniques. There are huge expectations for machine learning, and early signs point to its great potential for enzyme engineering in general and directed evolution in particular.

On the flip side, one of the biggest hurdles of bioinformatics is data availability. This approach works well on the large families of proteins for which a lot of sequence (and ideally functional) information is available but is less applicable for designing proteins from scratch or working with less diverse enzymatic families. Thus, far it is not clear if large improvements in activity can be obtained using this approach. A purely bioinformatic approach works best in the presence of at least some structural information (which is not hard to obtain these days) to prioritize the positions (e.g., around the active site) for directed evolution. Even the CBD approach can yield very large (possibly prohibitively large) number of mutants to test when distal sites are considered. Thus, sequence-based hot spot identification appears to be suited largely for fine-tuning properties of existing proteins and altering their specificities.

Data availability is a hurdle for machine learning as well. It does not work very well with small data sets. The spectacular success of AlphaFold was made possible by the wealth of information available through the PDB. Such well-ordered, uniformly described, high quality, and very diverse data sets do not exist for enzymes. On top of that, there is much higher experimental error associated with measurements of catalytic activities compounded by various ways of doing so (e.g., looking at kinetic parameters vs substrate turnover) and further influenced by conditions (pH, temperature, etc.). This is a major hurdle, yet not a fundamental one. With enough investment, large data sets of sufficient quality can be produced to develop algorithms capable of accurately representing such complex functions as enzymatic catalysis, a much bigger challenge.

## Dynamic Approaches to Mutagenic Hot Spot Predictions

**Figure 12:**
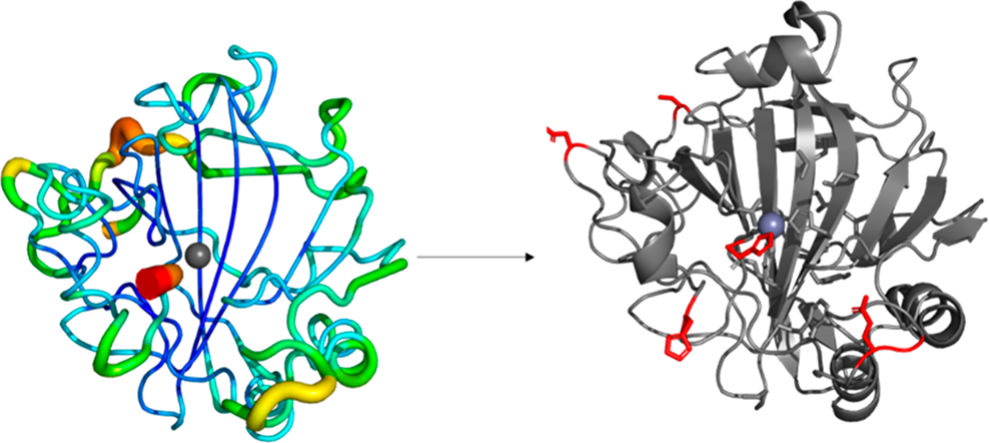
Overview of the dynamic approach to hot spot identification. Experimental dynamic information obtained using various experimental approaches to predict mutagenic hot spots.

Protein engineering for improving (thermo)stability has been done extensively, and in fact, the earliest directed evolution experiments were done to increase enzyme thermostability, as summarized in a recent outstanding review by Sun, Feng, Reetz, and co-workers.^55^ At the same time, there is a growing appreciation of the magnitude of dynamic effects on catalysis in general, as the structural approach alone does not fully describe all complexities of enzymatic catalysis. Thus, much recent effort has been dedicated to using protein dynamic information for mutagenic hot spot identification (Figure 12).

Historically, dynamic information for protein engineering has been obtained experimentally from analysis of crystallographic B-factors, which in turn necessarily requires X-ray crystal structures. Next, regions with high or low flexibility, depending on the desired outcome, are subjected to saturation mutagenesis, often with considerable success in increasing thermostability. Early approaches to use dynamic information were built on the notion that higher thermostability can be linked to improved catalytic efficiency. For example, Feng et al. explored dynamic effects for hot spot prediction in *Candida antarctica* lipase B (CalB). Two factors were considered for the identification of hot spots for further investigation: being within 10 Å of the catalytic Ser105 residue and high B-factor. A total of 6 residues were selected for iterative saturation mutagenesis. In addition, seven surface residues with high B-factor were chosen for saturation mutagenesis. *T*_50_^15^, the temperature at which 50% of enzyme activity remains after 15 min, was considered to evaluate kinetic stability. Two mutants (D223G and D278M) had *T*_50_^15^ increased by 2.4 and 3.8 °C, respectively, as compared to the wild type. Interestingly, combination of these two mutants (D223G/L278M) showed positive synergy in enhancing *T*_50_^15^ by about 12 °C over wild type. All three variants exhibited about 2–3 °C higher melting temperature than wild type, with no synergistic effect. Kinetic analyses were performed at 35 °C with all these variants toward *p*-nitrophenyl caprylate to determine catalytic parameters. Although the D223G mutant displayed a kinetic profile similar to wild type, both *k*_cat_ and *K*_M_ were higher for the double variant (D223G/L278M), resulting in similar catalytic efficiency (*k*_cat_/*K*_M_). However, L278M improves *k*_cat_/*K*_M_ by a factor of 2 over wild type, primarily dictated by an increase in *k*_cat_.^56^

**Figure 13:**
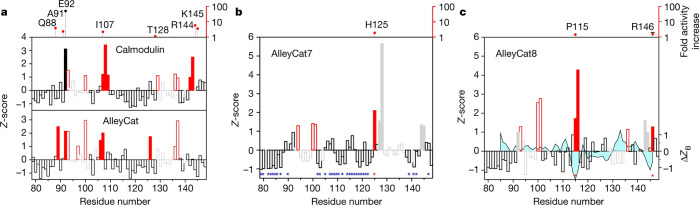
NMR derived hotspots in AlleyCat. Backbone amide chemical shift perturbation (CSP) upon addition of a transition state analog correlated with mutagenic propensities in the AlleyCat family of Kemp eliminases. Reproduced with permission from ref (62). Copyright 2022 Springer Nature.

Yet, the relationship between residue flexibility and catalysis in enzymes is complex, and rigidification of the active site residues and/or improving the thermostability of an enzyme may not necessarily be producing the desired outcome. The growing appreciation of the importance of the dynamic effects in enzymatic catalysis led us to investigate the applicability of nuclear magnetic resonance (NMR) for identification of mutagenic hot spots.^57−61^ NMR can provide residue specific dynamic information for soluble proteins of up to 50 kDa (and possibly more) under a variety of catalytically relevant conditions. The technique is rapid, and once residue assignments are made, a wealth of structural and dynamic information can be obtained. In fact, simple experiments such as chemical shift perturbation (CSP) in HSQC spectra of proteins offer critical snapshots of the change of the local microenvironment and dynamics upon a change in conditions, interaction with other molecules, etc. Using the functional information generated in directed evolution of AlleyCat, a calmodulin-based *de novo* designed enzyme designed to promote Kemp elimination, a model reaction for protein engineering studies, we retrospectively discovered a good correlation between CSP patterns upon addition of a transition state analog and location of productive mutations (Figure 13).^62^ Moreover, CSP maps allowed us to rapidly improve AlleyCat’s activity by a factor of 4 by identifying mutations away from the active site, when seemingly its evolutionary potential was exhausted. Interestingly, the CSP patterns were quite selective, i.e., no productive mutations were found in the regions that did not exhibit significant chemical shift perturbation. The chemical shift patterns upon addition of the transition state analog correlate well with the dynamic changes in the protein structure as demonstrated by B-factor analysis of the apo and inhibitor-bound crystal structures of the evolved variants, suggesting that the major determinants of the observed increase in the activity are of dynamic origin.

**Figure 14:**
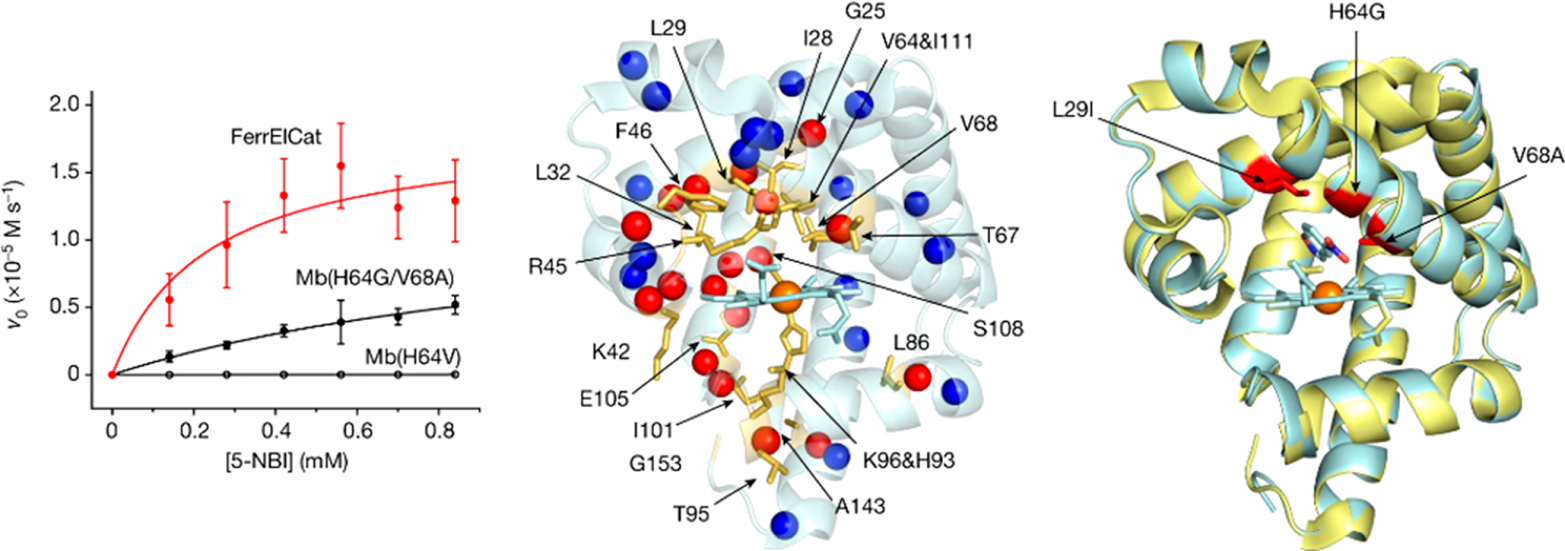
NMR-guided directed evolution of myoglobin. (Left) Michaelis–Menten plots for representative proteins. (Middle) NMR CSP data mapped on the X-ray crystal structure of Mb(H64V), showing the residues with prominent changes as yellow sticks. Spheres represent the residues with identified productive mutations (red) or those for which no productive mutations could be found (blue). (Right) Overlay of the crystal structures of Mb(H64V) (yellow) and FerrElCat with the docked inhibitor (cyan). The newly introduced mutations are shown in red. Reproduced with permission from ref (62). Copyright 2022 Springer Nature.

In a further test of the NMR-guided approach we applied it to myoglobin, a nonenzymatic protein. CSP analysis of Mb-H64V, a myoglobin mutant with nascent Kemp elimination activity using a redox-mediated mechanism, demonstrated a number of CSP hot spots (Figure 14).^63^ Saturation mutagenesis in every single one of those areas (except for one at the C-terminus, where soluble protein could be obtained for the screening hits) yielded productive mutations with improvement in catalytic efficiency, ranging from 2- to 71-fold (on average 20-fold). Saturation mutagenesis in the areas not showing significant CSP has not produced any productive mutations. With the use of a simplistic, nonexhaustive gene shuffling experiment, a combination of three residues identified in NMR-guided saturation mutagenesis experiments produced FerrElCat that shows catalytic efficiency of 1.5 × 10^7^ M^–1^ s^–1^, an improvement of 62,000-fold over Mb-H64V, the starting point for the directed evolution experiment. This level of activity is only 1–2 orders of magnitude away from the diffusion limit and highlights the power of the dynamic approaches to rapidly identify the mutagenic hot spots.

Overall, dynamic methods have been quite successful thus far. Even very straightforward evaluation of local flexibility improvements in catalytic efficiency are on par with those shown by bioinformatic approaches. Moreover, focusing on the changes in dynamics going to/from the transition states has shown some spectacular improvements. It remains to be seen how generalizable it can be and if/how other ways to bring in dynamic information (X-ray or H-D exchange-derived) can be used. Future work will also shed light onto how close the transition state analogs need to be to the transition state and how sensitive the approach is to it. Further experimental validation of this approach can result in rapid advance of ML approaches, as at least dynamic information can be obtained using already established tools.^64^ The principal limitation of the approach is data availability, and while NMR works well for smaller, soluble proteins, its applications for larger enzymes is limited.

## Conclusions

Where is directed evolution as a field now, and where do we go next? First, we must note that we view directed evolution as one of the approaches in the broader context of protein engineering (or design, depending on one’s personal preferences) since its ultimate goal is to make a protein with some desired property created or improved. To achieve that objective, directed evolution employs nature’s tools to do it efficiently, rapidly, and in a combinatorial fashion, as opposed to purely rational theoretical approaches. Yet, due to the fact that throughput of the screening approaches is (or very soon will be) on par with transformation efficiency, the brute force generation of even bigger libraries (produced at much cost) can be no match for the power function of the sequence search space, and the only way to advance the technique further is narrowing down the search space by identifying the locations where random or semirandom mutagenesis has a higher chance of yielding productive mutations.

In retrospect, given the fundamental numbers problem that we discussed in the introduction, it is quite stunning that epPCR has had so much success and is still being extensively used. In fact, Tawfik and others have estimated that up to 0.5% of all randomly introduced mutations into a protein can be functionally beneficial.^65^ From the practical perspective, it speaks volumes of the promise of the directed evolution as a whole. If there are so many possibilities out there to improve a given protein, then is the protein sequence space more functionally rich than originally thought? And the only tool we really need to take full advantage of is the more efficient approaches to search for the desired function.

Given the obvious success of directed evolution in various practical applications, it is a bit disheartening to see that the number of approaches to limit the search space, clearly the best way to help realize its full potential, is still fairly small, naïve, and only mostly employs very basic concepts, such as binding pocket reshaping, sequence alignment, etc. Combining them in different ways does provide good and very usable practical outcomes, yet still it is more art than science. While Reetz and his ISM approach came the closest to a systematic approach, we are still quite far away even from realizing the full potential of the structure-based hot spot identification let alone the fully fledged protein space exploration. Yet, recent work on focusing on enzyme dynamics and machine learning has the potential to greatly advance the efficiency of hot spot prediction. Machine learning shows immense promise and outperforms human judgements even in fairly simple data sets.^66^ There is every reason to believe, if we were ultimately to successfully solve the problem of creating efficient catalysts using ML, artificial enzymes would be the first space to do so, just due to the sheer number of data already generated, common language of protein sequences (as opposed to the complexity of organic molecules as a whole), and outstanding ability to perform structure predictions using already available tools. Yet, there are quite a few challenges on that road: one, as described above, is the amount of functional data and its quality. Even in larger families the amount of functional data obtained under similar conditions may not be enough for ML approaches to reach their full potential. Additionally, unlike structural data banks, functional information may be of much lower quality, poor data reproducibility is not unheard of in the field, and moreover, fundamentally different types of characterization (e.g., *k*_cat_ vs TON) obtained under a variety of conditions can be reported in different cases. Yet fundamentally it is certainly feasible to generate large enough and diverse enough functional data sets at least in some cases to jump start algorithm development that can lead to rapid development of ML for protein engineering. This is definitely the field to watch in the next decade.

Improving methods for directed evolution have also significant implications for creating enzymes from scratch. Most early directed evolution work dealt with improving properties of existing enzymes and repurposing them to other substrate or mechanistically similar chemical transformations. Yet, to be practical, directed evolution requires some reasonable starting point. Over the past few decades, much effort has been devoted to the design of enzymes for practically important chemical reactions from scratch, or *de novo*. Indeed, the lure of the ability to take advantage of nature’s tools to promote practical transformations with no known natural analogs has driven development of quite a few different protein design approaches. Yet, all of them failed to produce highly active enzymes even for relatively simple model reactions despite formidable resources used.^67^ Only after multiple rounds of extensive directed evolution could they be (in few cases) evolved to show truly enzymatic levels of efficiency.^68^ The apparent failure of the design field was not for the lack of trying: many well-funded groups at top institutions all over the world spent decades on the problem. However, our collective inability to go from retrospective, seemingly pretty good, deciphering of how enzymes operate to prospectively create a least one good one, given the excellent structure prediction tools at our disposal, demonstrates that our understanding of the protein function is far from complete. Indeed, as Richard Feynman famously noted: ‘What I cannot create – I do not understand’. In this regard, directed evolution serves two purposes: from the practical standpoint it allows for rapid experimental improvement of catalytic efficiency of the modestly active enzymes generated by the present-day computational approaches. Further improvement of directed evolution approaches should favor the minimalist approach to protein design. If large improvements in catalytic efficiency in promoting unnatural chemical reactions can be obtained in designed enzymes based on nonenzymatic proteins such as calmodulin and myoglobin then perhaps protein evolvability is much broader than previously thought?^69,70^ Intuitively it makes sense, as despite the fairly small number of protein folds (estimated to be in the low thousands or less) nature has developed a myriad of different enzymes capable of amazing chemistry.^71^ Perhaps the specialization we observe in some enzyme superfamilies is not a function of the fold itself but rather a product of evolution, starting from a particular (possibly random) starting point? This is also supported by the fact that some of the activities that are absolutely essential for carbon-based life (e.g., carbon dioxide hydration) are promoted by enzymes that have drastically different overall folds in various organisms.^72^ If this is true then rapid computational placement of primitive active sites with a bare minimum functionality into various protein scaffolds to identify possible starting points followed by highly efficient directed evolution may constitute a more practical approach to *de novo* enzyme design that extensively focuses on faithful replication of transition state geometry in the lowest energy state protein fold.^46,73−76^

Last but not least, directed evolution has been incredibly useful in improving basic fundamental understanding of enzymatic functions. It provided a much better, albeit clearly not fully complete, picture of the contributions of stability, evolvability, dynamic, and steric effects to enzymatic catalysis.^61,77,78^ It helps to better understand the folding and functional fitness landscape and offers an insight into protein evolution and design.^79−82^ Narrowing down the number of combinations in directed evolution experiments comes hand-in-hand with fundamental understanding of the enzymatic function, and in a way, directed evolution is being evolved itself.
